# Neural substrates of propranolol-induced impairments in the reconsolidation of nicotine-associated memories in smokers

**DOI:** 10.1038/s41398-021-01566-6

**Published:** 2021-08-24

**Authors:** Xiao Lin, Jiahui Deng, Kai Yuan, Qiandong Wang, Lin Liu, Yanping Bao, Yanxue Xue, Peng Li, Jianyu Que, Jiajia Liu, Wei Yan, Hongqiang Sun, Ping Wu, Jie Shi, Le Shi, Lin Lu

**Affiliations:** 1 Peking University Sixth Hospital, Peking University Institute of Mental Health, NHC Key Laboratory of Mental Health (Peking University), National Clinical Research Center for Mental Disorders (Peking University Sixth Hospital), Chinese Academy of Medical Sciences Research Unit (No. 2018RU006), Peking University, 100191 Beijing, China; 2 Beijing Key Laboratory of Applied Experimental Psychology, National Demonstration Center for Experimental Psychology Education (Beijing Normal University), Faculty of Psychology, Beijing Normal University, 100875 Beijing, China; 3 National Institute on Drug Dependence and Beijing Key Laboratory on Drug Dependence Research, Peking University, 100191 Beijing, China; 4 Peking-Tsinghua Center for Life Sciences and PKU-IDG/McGovern Institute for Brain Research, 100191 Beijing, China

**Keywords:** Learning and memory, Psychiatric disorders

## Abstract

The majority of smokers relapse even after successfully quitting because of the craving to smoking after unexpectedly re-exposed to smoking-related cues. This conditioned craving is mediated by reward memories that are frequently experienced and stubbornly resistant to treatment. Reconsolidation theory posits that well-consolidated memories are destabilized after retrieval, and this process renders memories labile and vulnerable to amnestic intervention. This study tests the retrieval reconsolidation procedure to decrease nicotine craving among people who smoke. In this study, 52 male smokers received a single dose of propranolol (*n* = 27) or placebo (*n* = 25) before the reactivation of nicotine-associated memories to impair the reconsolidation process. Craving for smoking and neural activity in response to smoking-related cues served as primary outcomes. Functional magnetic resonance imaging was performed during the memory reconsolidation process. The disruption of reconsolidation by propranolol decreased craving for smoking. Reactivity of the postcentral gyrus in response to smoking-related cues also decreased in the propranolol group after the reconsolidation manipulation. Functional connectivity between the hippocampus and striatum was higher during memory reconsolidation in the propranolol group. Furthermore, the increase in coupling between the hippocampus and striatum positively correlated with the decrease in craving after the reconsolidation manipulation in the propranolol group. Propranolol administration before memory reactivation disrupted the reconsolidation of smoking-related memories in smokers by mediating brain regions that are involved in memory and reward processing. These findings demonstrate the noradrenergic regulation of memory reconsolidation in humans and suggest that adjunct propranolol administration can facilitate the treatment of nicotine dependence. The present study was pre-registered at ClinicalTrials.gov (registration no. ChiCTR1900024412).

## Introduction

Nicotine addiction is a chronic relapsing disorder and one of the leading causes of preventable death worldwide [1]. During the 20th century, 100 million people died from tobacco exposure [2]. Repeated contiguous pairings between smoking-related cues and nicotine reinforcement lead to the cues acquiring the ability to elicit a host of conditioned responses (e.g., subjective craving) [3–5]. As the state of addiction develops, the processing of smoking-related cues becomes controlled by automatic stimulus–response associations via the abnormal activation of mesocorticolimbic circuitry. Associative learning and memory processes are contributory causal factors in the establishment and maintenance of nicotine-reinforced smoking. If the maladaptive memories for this learning can be disrupted, then their contribution to the maintenance of smoking behavior can be reduced or eliminated, thereby decreasing the risk of relapse in smokers attempting to quit [6, 7].

The reconsolidation hypothesis describes that memories, when reactivated, enter a transient, labile state followed by a re-stabilization termed reconsolidation [8, 9], which may provide an opportunity to destabilize or erase established maladaptive memories [10, 11]. Preclinical studies revealed that the systematic administration of protein synthesis inhibitors within the reconsolidation time window led to the absence of expression of maladaptive memories [12–14]. However, most amnesic agents that are tested in animals are unsuitable for use in humans. Propranolol, an antagonist of β-adrenergic receptors, has wide clinical applications. Propranolol inhibits norepinephrine-stimulated cyclic adenosine monophosphate response element-binding protein phosphorylation, thereby inhibiting indirectly protein synthesis [15, 16]. Most previous studies focused on disrupting the reconsolidation of emotional memory in healthy participants [17] and patients with posttraumatic stress disorder [18–22]. More recent studies have investigated reconsolidation as a therapeutic target for drug addiction and found that propranolol significantly reduced both subjective craving and psychophysiological arousal (i.e., heart rate and blood pressure) in response to drug-related cues [23–26]. Our early study found that propranolol administration in abstinent heroin addicts before the reactivation of a word list impaired the reconsolidation of heroin-related words [27]. We also found that propranolol administration before memory retrieval decreased craving in smokers [28]. However, neuroimaging evidence of the mechanisms by which propranolol interferes with the reconsolidation of nicotine-related memories remains scarce.

Previous functional magnetic resonance imaging (fMRI) studies suggested that interrupting the reconsolidation of fear memories mainly alters the activation of the prefrontal cortex, amygdala, and hippocampus. These three brain regions mediate emotional responses to cues and memories. For example, behavioral extinction during reconsolidation decreased involvement of the prefrontal cortex, together with the amygdala and hippocampus [29–32]. Using functional brain imaging in individuals with a lifelong fear of spiders, a previous study showed that fear memory was activated by repeated exposure to feared cues, and activity in the basolateral amygdala was decreased at re-exposure 24 h later [33]. The combination of propranolol and exposure therapy to reduce fear memory mainly reduced activation of the medial prefrontal cortex (mPFC) and enhanced activation of the hippocampus [34]. Thus, we hypothesized that impairment of the associations between smoking-related cues and nicotine induced by propranolol during reconsolidation decreases the reactivity of brain regions that are related to cue processing and mediate the activation of memory-related brain regions. Neuroimaging data (fMRI) were collected during the memory reconsolidation process and during a cue-reactivity paradigm 1 day before and 1 day after the reconsolidation manipulation.

## Materials and methods

### Participants

Fifty-two healthy male smokers were enrolled in the study through advertisements, which encourages motivated smokers to enroll. The inclusion criteria for smokers were the following: (1) right-handed and (2) smoking eight or more cigarettes per day over the past year or Fagerstrom Nicotine Dependence Test (FNDT) score ≥4. Participants were excluded if they (1) had cardiovascular diseases, (2) had used addictive drugs other than nicotine, (3) current or past history of medical or psychiatric illness, diagnosed by the Structured Clinical Interview for Diagnostic and Statistical Manual of Mental Disorders, 4th edition, Axis I Disorders (SCID). It is widely known that anxiety may affect the craving to smoking in smokers, (4) were currently using antidepressants or had clinically evident cognitive impairment, or (5) took any prescription drug during the 2 weeks before the experimental sessions. The participants were free from fMRI contraindications and propranolol contraindications (e.g., bronchial asthma, cardiogenic shock, heart block [II to III degree atrioventricular block], severe heart failure, or sinus bradycardia). All of the participants were instructed to refrain from drug, alcohol, and caffeine consumption for 24 h before the experiment, and they were suggested not to smoke 3 h before arriving in the laboratory. However, the assessments of substance use relied exclusively on self-report abstinence. The sample size was determined a priori using GPower 3.1.9. Calculations revealed, based on a two-tailed *α* = 0.05, *β* = 0.20, and *ρ* = 0.50 that 26 participants would be required. Each participant provided written informed consent and was paid for participation in the study. The study protocol was approved by the Institutional Review Board of Peking University Sixth Hospital.

The qualified participants were randomly and blindly assigned to the propranolol group or placebo group in a 1:1 ratio using simple randomization with computer-generated random numbers. Each participant was required to attend the study for 3 consecutive days. Fifty-two smokers completed the 3 days of evaluation (*n* = 27 in the propranolol group, *n* = 25 in the placebo group). Four participants in the propranolol group and one participant in the placebo group did not finish the cue-reactivity task in the fMRI scanner on day 3, thus leaving 47 participants (*n* = 23 in the propranolol group, *n* = 24 in the placebo group) whose neuroimaging data from cue-reactivity task were analyzed.

### Procedure

**Fig. 1:**
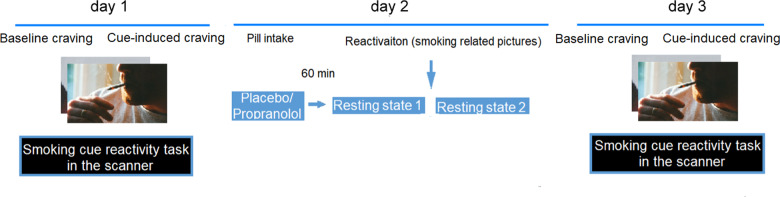
Experimental design and protocol. Recruited smokers came to the lab, completed the craving evaluation, and performed a cue-reactivity task in the fMRI scanner. Twenty-four hours later, they took a placebo or propranolol (40 mg) pill 1 h before they underwent two resting-state fMRI scans. Before the second scan, the participants reactivated the nicotine-associated memories by being exposed to smoking-related pictures. Again 24 h later, all of the participants completed the craving evaluation and the same cue-reactivity task in the scanner.

The experimental procedure is shown in Fig. 1. All of the participants were required to come to the laboratory for 3 consecutive days. On the first day, they were briefed on the study protocol and requirements and completed a demographic questionnaire and other scales, including the FNDT, Beck Depression Inventory (BDI), Self-rating Anxiety Scale (SAS), and Barratt Impulsivity Scale (BIS). These scales are set to exclude the potential effect of impulsivity on the procedure, which have been reported to affect the brain reactivity to smoking-related cues in smokers. Each participant graded their level of smoking craving on a visual analog scale, marked from 0 on the left (“extremely low”) to 10 on the right (“extremely high”). They graded their level of craving again after exposure to smoking-related pictures outside of the scanner, which was considered preexisting conditioned cue-induced craving for smoking. The participants completed the cue-reactivity task in the fMRI scanner to measure their baseline brain reactivity to smoking-related pictures. In each trial of the cue-reactivity task, three pictures appeared on the screen for 2 s, with one at the top of the screen and two at the bottom. The participants were required to press a key (left key or right key) to indicate which picture on the bottom was the same as the picture on the top. There were 24 smoking-related pictures and 24 neutral pictures, and all of the pictures contain a similar framework except for the neutral picture, the cigarettes are replaced by a pen or something similar. Each picture was presented twice in the scanner, for a total of 96 trials. The variable ISIs were designed as the jitter (500–1500 ms) required to isolate the hemodynamic response to each stimulus necessary in an event-related fMRI design. For each trial, the reaction time was limited to 4 s. Once the participant made a response, the screen went black until 4 s elapsed for each trial, and this procedure was set up to make sure the times are consistent across trials and participants. Timeouts were not included in the statistical analysis. The participants completed the same task on day 3 to investigate the effect of reconsolidation manipulation (day 2) on brain reactivity to smoking-related pictures/cues.

On day 2, the participants received a placebo pill (20 mg vitamin C) or propranolol pill (40 mg, p.o.; YABANG Pharma, *propranolol HCl, immediate-release formulation*), depending on group assignment. All of the pills were packed in colorless and odorless capsules to avoid drug detection induced by the taste of the drugs. Vitamin C has been used as a placebo in previous studies [35, 36]. To verify the pharmacological action of propranolol, heart rate measurements were taken immediately before and 60 min after administration. The participants were not told about their heart rates. The participants then underwent the first resting-state fMRI scan (8 min). Afterward (~70 min after drug intake), the participants were presented with the same smoking-related pictures one by one (24 pictures, 5 s presentation per picture, for a total of 2 min) that were presented on day 1 and told to view them freely. The purpose of presenting these smoking-related pictures was to reactivate smoking-related memories, thus activating memory reconsolidation. Immediately after the reactivation procedure, the participants underwent the second resting-state fMRI scan to determine the effect of propranolol on brain activity during memory reconsolidation. The 70-min interval between drug intake and reactivation is consistent with previous studies [29, 37], which also confirms the pharmacodynamic effects of propranolol in humans [38], such that memory reactivation occurred during peak propranolol levels.

### fMRI data acquisition

A GE-MR750 3.0 Tesla scanner was used to acquire images at the MRI Research Center, Peking University. The scanning included functional and anatomical imaging. T2*-weighted functional images were acquired in 40 axial slices that were parallel to the AC–PC line with no interslice gap, thus affording full-brain coverage. Functional images were collected using an echo-planar imaging sequence (33 axial slices, 4.2-mm slice thickness, TR = 2000 ms, TE = 30 ms, 3.5 × 3.5 × 4.2 mm voxel size, 90° flip angle, 224 × 224 mm field of view). Structural images were acquired using a three-dimensional sagittal T1-weighted magnetization-prepared rapid gradient echo (192 slices, 1-mm slice thickness, 1 × 1 × 1.0 mm voxel size, 12° flip angle, 450-ms inversion time, 256 × 256 mm field of view).

### fMRI data preprocessing and analysis

Statistical Parametric Mapping v. 8 software (Wellcome Trust Department of Cognitive Neurology, London, UK) was used to preprocess the image data. Images were analyzed using standard preprocessing procedures (slice time-corrected, motion-corrected, resampled to 3 × 3 × 3 isotropic voxels, normalized to Montreal Neurological Institute space, spatially smoothed using a 6-mm FWHM Gaussian filter, and temporally filtered using a high-pass filter with 1/120 Hz cutoff frequency). The data analysis was conducted using the MATLAB-based NeuroElf toolbox (https://neuroelf.net/). Motion time courses were obtained by estimating the values for translation (mm) and rotation (degree) for each subject. The participants who had more than 2 mm maximum displacement in *X, Y*, or *Z* and 2° of angular motion in all rfMRI scans were rejected [39–41].

In a previous study, Schwabe et al. used a similar study protocol and chose to conduct ANOVA at different time point to get clearer results [29]. To analyze the brain reactivity to smoking-related cues, we conducted a two-way ANOVA at baseline, with a group (propranolol, placebo) as the between-subjects factor and cue type (smoking-related cues, neutral cues) as the within-subjects factor on day 3, and this procedure is similar to a previous study [29]. To further identify propranolol-induced changes in brain reactivity to smoking-related cues during reconsolidation, we conducted a time (day 1, day 3) × group (propranolol, placebo) ANOVA of brain reactivity to smoking-related cues. First-level effects were carried out by means of general linear models, incorporating task effects and covariates of no interest (a linear trend to account for low-frequency drift and rigid-body motion parameters were included as single-subject regressors to partially account for movement effects, age, and education) were convolved with a canonical hemodynamic response function and used to compute parameter estimates (*β*) and contrast images (containing weighted parameter estimates) for each cue category at each voxel. Contrast images for each subject, comparing smoking vs. neutral scenes were entered into a second-level, random-effects analysis with participant treated as the random effect. Monte Carlo simulations using AlphaSim were used to calculate the minimum cluster size at a whole-brain correction of *P* < 0.05. Simulations (1000 iterations) were performed and resulting in a minimum cluster size of 38 contiguous voxels.

In order to test the hypothesis that severity of smoking dependence is related to activity in the brain regions involved in smoking cue processing, Pearson correlations were carried out between BOLD signals in clusters that showed a significant difference in response to smoking cues compared with neutral cues and the severity of smoking dependence (FNDT).

To investigate the neural mechanisms that underlie the effects of propranolol on the reconsolidation process, we compared resting-state brain activity, revealed by fMRI, before and after exposure to smoking-associated pictures in the propranolol and placebo groups. Using the bilateral hippocampus as a region of interest, differences in functional connectivity were analyzed between groups. Data of the resting-state fMRI on day 2 from two participants from the placebo group because of excessive head motions during scanning. The results were corrected for multiple comparisons using the same method (Alphasim corrected *P* < 0.05, cluster >38). To test that the increased functional connectivity is correlated with the effect of reconsolidation manipulation, brain regions that presented differences in functional connectivity to the hippocampus were extracted to investigate relationships with craving scores on day 3.

## Results

### Disruption of reconsolidation by propranolol decreased nicotine craving

**Fig. 2:**
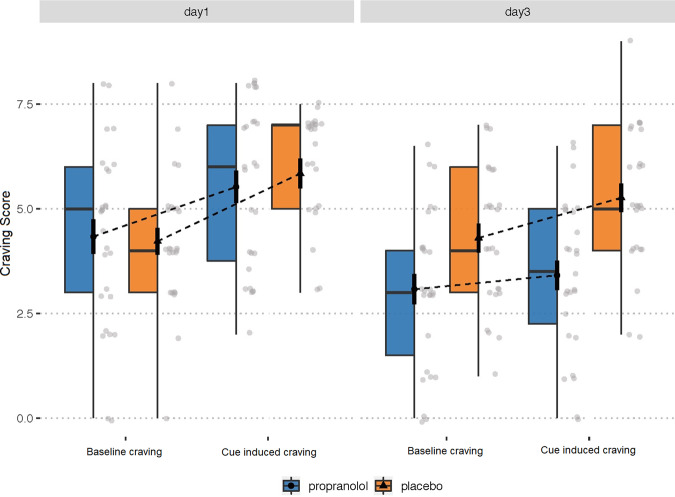
Propranolol-induced disruption of reconsolidation decreases smoking craving. The ANOVA showed a significant group (propranolol, placebo) × time (day 1, day 3) interaction for craving scores. The simple-effect analysis revealed that craving scores significantly decreased from day 1 to day 3 in the propranolol group but not in the placebo group. This analysis also revealed that after the reconsolidation manipulation, the propranolol group (blue bars) exhibited a more significant decrease compared with the placebo group (orange bars). Error bars indicated the SEM.

Independent-sample *t* tests indicated no significant differences between the propranolol and placebo groups in age, body mass index, years of education, FNDT scores, BDI scores, SAS scores, and BIS scores (all *P* > 0.05; Table 1). Participants were suggested not to smoke 3 h before arriving in the laboratory. The expired air was analyzed for CO in parts per million (ppm) by using a CO detector (Bedfont Mini2 Smokerlyzer, Bedfont Scientific Ltd.), and expired air CO levels showed no significant difference between the two groups (propranolol: 5.19 ± 3.12, placebo: 4.25 ± 3.05) at baseline. The hypothesis is that there is a group and time interaction effect on craving score. To analyze the changes of craving score, we conducted a three-way (group as between-subject factor, time and cue exposure as within-subject factors) ANOVA, which showed a significant group (propranolol, placebo) × time (day 1, day 3) interaction for craving scores (*F*(1,50) = 9.55, *P* = 0.003; *η*² = 0.162, observed power = 0.86, Fig. 2). The simple-effect analysis revealed that craving scores significantly decreased from day 1 to day 3 (*P* < 0.001) in the propranolol group but not in the placebo group (*P* = 0.46). This analysis also revealed that after the reconsolidation manipulation, craving for smoking significantly decreased in the propranolol group compared with the placebo group, baseline and cue-induced craving (see Fig. 2). These results indicate that propranolol disrupted smoking memory reconsolidatio by which it decreased craving to smoking. It is worth noting that we also detected a significant decrease in baseline craving on day 3.

**Table 1:** Demographic data and psychological traits of the participants in each group.

Characteristic	Propranolol	Placebo	*P*
	*n* = 27	*n* = 25	
	Mean (SD)	Mean (SD)	
Age (years)	27.89 (6.69)	28.24 (7.94)	0.83
Education (years)	14.38 (2.73)	13.79 (1.44)	0.40
BMI	23.78 (3.34)	22.33 (2.94)	0.49
Age when first smoked	18.69 (3.0)	19 (3.5)	0.74
Duration of smoking (years)	9.63 (6.95)	8.96 (7.33)	0.72
Cigarettes per day	13.4 (6.1)	15.1 (8.4)	0.48
FNDT score	5.08 (1.38)	5.48 (1.81)	0.37
BDI score	4.19 (4.85)	5.08 (6.56)	0.58
SAS score	32.92 (8.48)	33.16 (8.33)	0.92
BIS score	47.92 (17.48)	48.64 (14.49)	0.87

*BMI* body mass index, *BDI* Beck Depression Inventory, *FNDT* Fagerstrom Nicotine Dependence Test, *SAS* self-rating Anxiety Scale, *BIS* Barratt Impulsivity Scale.

The data are expressed as mean ± standard deviation.

### Neural correlates of the propranolol-induced disruption of reconsolidation

**Fig. 3:**
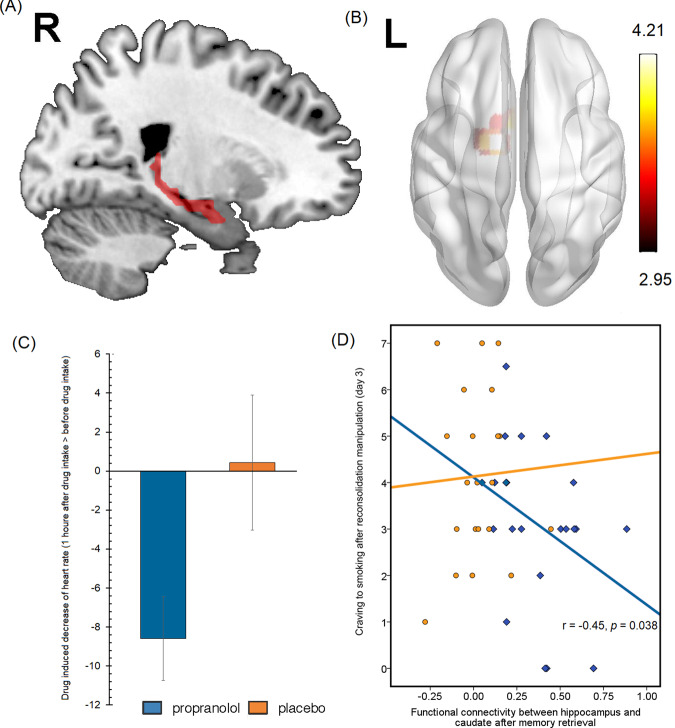
Increase in functional connectivity between the hippocampus and striatum in the propranolol group during the reconsolidation process. **A**, **B** The hippocampus as the region of interest and brain regions that showed an increase in connectivity with the hippocampus in the propranolol group during the second resting-state fMRI scan. **C** Propranolol administration significantly decreased heart rate. **D** Functional connectivity between the hippocampus and striatum negatively correlated with craving scores on day 3 in the propranolol group. No significant correlation was detected in the placebo group.

We next sought to identify brain regions that were involved in impairments in reconsolidation that were induced by propranolol. A significant decrease in heart rate and blood pressure was observed after propranolol administration, thus confirming the pharmacological effect of propranolol (*t*_50_ = 2.21, *P* = 0.03 for heart rate, *t*_50_ = 2.78, *P* < 0.01 for systolic pressure, *t*_50_ = 4.76, *P* < 0.01 for diastolic pressure; Fig. 3C). After exposure to smoking-related pictures on day 2, the propranolol group exhibited greater functional connectivity between the hippocampus and striatum than the placebo group (Fig. 3A, B). We conducted a functional connectivity analysis using the hippocampus as the seed on the resting-state data and found no significant difference between the two groups on day 1. We also analyzed the functional connectivity differences between two groups before exposure to smoking-associated pictures and found no significant difference in hippocampus connectivity. The functional connectivity during reconsolidation was negatively correlated with craving scores on day 3 in the propranolol group (Fig. 3D), suggesting a correlation between higher functional connectivity between the hippocampus and striatum during the reconsolidation window and lower craving for smoking on day 3. Furthermore, the increase in functional connectivity between the striatum and hippocampus was only detected during the second resting-state scan, meaning that propranolol did not affect functional connectivity between the hippocampus and striatum without memory reactivation. These results were similar to a previous study that investigated emotional memory and focused on the hippocampus as the region of interest, which also found an increase in activity in the hippocampus in the propranolol and retrieval group [29].

### Alterations of brain reactivity to smoking-related cues after the propranolol-induced disruption of reconsolidation

**Fig. 4:**
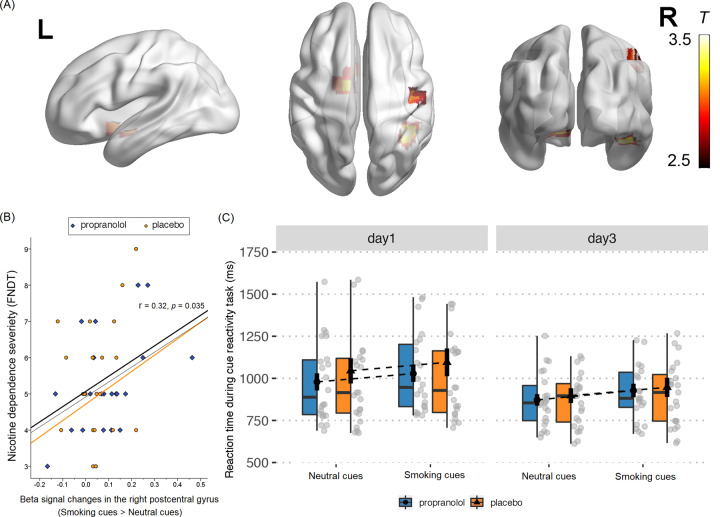
Brian reactivity to smoking-related cues at baseline (day 1). **A** Brain regions that showed a significant increase in activation in response to smoking-related cues at baseline (smoking-related cues > neutral cues). The peak brain regions were the caudate and postcentral gyrus. **B** A positive correlation was found between the increase in postcentral gyrus activity and the severity of nicotine dependence, measured by FNDT scores. **C** Reaction time during the cue-reactivity paradigm, with a main effect of cue type (i.e., the reaction time for smoking-related cues was longer than for neutral cues) and time (i.e., the reaction time on day 3 was shorter than on day 1).

We further tested whether this reconsolidation manipulation impacts brain activity that is involved in cue reactivity. To examine baseline brain reactivity to smoking-related pictures in smokers, a cue-reactivity task was performed in the fMRI scanner. At baseline, no effect of group on brain reactivity to smoking-related cues was found at baseline, so we pooled the data to achieve a clear neural signature of brain reactivity to smoking-related cues. The striatum and postcentral gyrus exhibited an increase in activation in response to smoking-related cues compared with neutral cues (Fig. 4A and Table 2). We also found that the beta signal in the postcentral gyrus showed a significant positive correlation with the severity of nicotine dependence (i.e., FNDT scores) on day 1 (Fig. 4B). We also did a three-way ANOVA on the neuroimaging data of the cue-reactivity task (see Supplementary Figs. S1 and S2 and Supplementary Tables S1 and S2).

**Table 2:** Clusters that showed a significant increase in activation in response to smoking-related cues at baseline (smoking-related cues > neutral cues).

Anatomical region	Coordinates			*T*	Cluster size (no. of voxels)	Hemisphere
	*X*	*Y*	*Z*			
Midbrain subthalamic nucleus (caudate)	−9	−8	−3	3.14	125	L
Postcentral gyrus	48	−20	44	2.94	140	R
Declive	48	−49	–21	3.45	140	R
Inferior semi-lunar lobule	−24	−61	−42	3.2	41	L

Coordinates are given for the maximally significant voxel in each area, where *X* defines the lateral placement from the midline (left = negative), *Y* defines the anteroposterior displacement relative to the anterior commissure (posterior = negative), and *Z* defines the vertical position relative to the anteroposterior commissural line (down = negative). *P* values were corrected for multiple comparisons at *P* < 0.001 (Monte Carlo simulations using AlphaSim were used to calculate the minimum cluster size at an uncorrected threshold of *P* < 0.001 required for a whole-brain correction of *P* < 0.05. Simulations (1000 iterations) were performed and resulting in a minimum cluster size of 38 contiguous voxels.). The coordinates are in Montreal Neurological Institute (MNI) space. The brain regions were automatically identified by NeuroElf software.

**Fig. 5:**
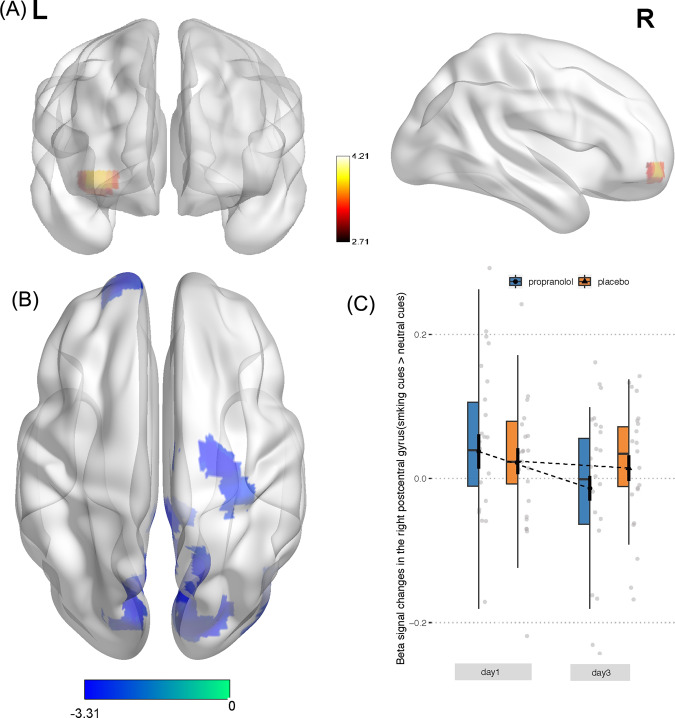
Alteration of brain reactivity to smoking-related cues after the reconsolidation manipulation. **A** Brain regions that showed an increase in activation in response to smoking-related cues compared with neutral cues in the propranolol group on day 3 (propranolol > placebo). **B** Brain regions that showed a decrease in activation in response to smoking-related cues on day 3 compared with day 1 in the propranolol group (propranolol > placebo). **C** Decrease in reactivity of the postcentral gyrus to smoking-related cues after the reconsolidation manipulation in the propranolol group.

To analyze the behavior response to smoking-related cues, we conducted a three-way ANOVA of reaction time during the cue-reactivity task, which revealed a significant main effect of time (day 1, day 3: *F*(1,45) = 12.50, *P* < 0.001, *η*² = 0.22, observed power = 0.93) and the main effect of cue type (smoking-related cues, neutral cues: *F*(1,45) = 31.11, *P* < 0.001, *η*² = 0.41, observed power = 1.00), but no effect of group (Fig. 4C). The lack of a difference between groups might be attributable to a floor effect, in which the reaction time was too short to detect a group difference. To directly compare differences in brain reactivity to smoking-related cues in the two groups on day 3, we conducted a two-way ANOVA, with the group as the between-subjects factor and cue type as the within-subjects factor. A significant group × cue type interaction was found in the mPFC, with an increase in activation of the mPFC in response to smoking-related cues compared with neutral cues in the propranolol group, compared with the placebo group (Fig. 5A and Table 3). To further reveal longitudinal changes in brain reactivity to smoking-related cues from day 1 to day 3, another ANOVA of brain reactivity to smoking-related cues was conducted, with the group as the between-subjects factor and time as the within-subjects factor. This analysis revealed a decrease in the involvement of the postcentral gyrus with smoking-related cues in the propranolol group compared with the placebo group (Fig. 5B, C and Table 3). As mentioned above, the reactivity of the postcentral gyrus to smoking-related cues was positively correlated with the severity of nicotine dependence. This decrease in reactivity suggested successful impairment of memories of the association between smoking-related cues and nicotine.

**Table 3:** Alterations of brain reactivity to smoking-related cues after the reconsolidation manipulation.

Anatomical region	Coordinates			*T*	Cluster size (no. of voxels)	Hemisphere
	*X*	*Y*	*Z*			
Smoking > neutral cues (propranolol > placebo)						
Middle frontal gyrus	−20	−6	37	3.31	53	L
Middle frontal gyrus	42	52	−4	3.15	29	R
Smoking day 3 > smoking day 1 (propranolol > placebo)						
Precentral gyrus	32	−14	67	−3.22	50	R
Posterior cingulate	9	−38	12	−2.98	38	R
Cuneus	19	−77	19	−3.04	260	R
Cuneus	−13	−81	26	−3.02	132	L
Middle occipital gyrus	53	−73	1	−2.94	42	R
Superior frontal gyrus	−19	63	15	−2.92	70	L
Postcentral gyrus	31	−35	49	−2.91	52	R

Coordinates are given for the maximally significant voxel in each area, where *X* defines the lateral placement from the midline (left = negative), *Y* defines the anteroposterior displacement relative to the anterior commissure (posterior = negative), and *Z* defines the vertical position relative to the anteroposterior commissural line (down = negative). *P* values were corrected for multiple comparisons (for the Smoking > neutral cues (propranolol > placebo) on day 3, AlphaSim corrected *P* < 0.05, cluster size k-threshold of 24 contiguous voxels at uncorrected threshold *P* < 0.001; for the Smoking day 3 > smoking day 1 (propranolol > placebo), AlphaSim corrected *P* < 0.05, cluster size *k*-threshold of 38 contiguous voxels at uncorrected threshold *P* < 0.001). The coordinates are in Montreal Neurological Institute (MNI) space. The brain regions were automatically identified by NeuroElf software.

## Discussion

This study investigated the effects of propranolol on the reconsolidation of smoking memories. We observed a decrease in craving and a concomitant decrease in postcentral gyrus reactivity to smoking-related cues in smokers after the pre-retrieval manipulation of reconsolidation by propranolol. Brain regions that are involved in reward memory exhibited propranolol-induced changes in activation after memory retrieval. Functional connectivity between the hippocampus and striatum during the reconsolidation time window increased in the propranolol group compared with the placebo group. These findings indicate that pre-retrieval propranolol administration blocked the re-stabilization of smoking-related cues.

Previous neuroimaging studies sought to reveal the neural mechanisms of drug-related cue-induced craving and relapse in nicotine addiction. A quantitative meta-analysis found that the anterior cingulate cortex, mPFC, and ventral striatum were related to smoking cue-reactivity and self-reported craving, suggesting that these brain regions constitute a core circuit of drug craving in nicotine addiction [42]. Although the hypothesis is that the memory circuit, especially the brain regions involved in memory retrieval, is related to smoking cue reactivity, most of the reported brain regions are concentrated upon mesocorticolimbic system, as discussed in the previous review [43]. This is partially consistent with this study, in which smokers exhibited an increase in activation of the striatum in response to smoking-related cues at baseline. The striatum is one of the most essential regions of the reward system. It plays an important role in the initiation of drug-seeking behavior in response to drug cues and may thus reflect a process that exaggerates the value of smoking-related cues. Another study reported that subjective craving for smoking correlated with an increase in activity in both reward-related brain structures and the postcentral gyrus [44]. We also found that smoking-related cues increased activation in the postcentral gyrus, and this increase was positively correlated with the severity of nicotine dependence (FTND). In addition, after the disruption of reconsolidation, reactivity of the postcentral gyrus to smoking-related cues significantly decreased. These results indirectly indicate disruption of the association between smoking-related cues and nicotine. The reconsolidation manipulation also increased reactivity of the mPFC to smoking-related cues. A previous study investigated correlations between activation of the mPFC in response to smoking-related cues and craving, and found a correlation between an increase in mPFC activation and resistance to smoking [45]. Reactivity of the mPFC to smoking-related cues may reflect a decrease in the association between smoking-related cues and smoking itself and greater inhibition of smoking urges. We failed to detect neural changes in the amygdala. This could be caused by the attributes of the smoking-related pictures, which have high reward value while low emotional valence. The smoking-related pictures induced the increased activation of the striatum while no change of the amygdala was detected.

Brain reactivity to smoking-related cues can only provide indirect evidence of the effects of reconsolidation manipulation. To further explore the effect of propranolol on memory reconsolidation, we compared brain activity after retrieval between the propranolol and placebo groups. We found an increase in functional connectivity between the hippocampus and striatum after memory reactivation in the propranolol group. This result appears to contrast with the notion that propranolol impairs memory reconsolidation, in which a decrease in activation of memory-related brain regions should be observed. Schwabe et al. also reported an increase in activation of the hippocampus in the propranolol group when memory was retrieved by related cues [29]. Norepinephrine has been shown to increase memory consolidation by acting on β-adrenergic receptors, which stimulates the cyclic adenosine monophosphate-dependent protein kinase pathway [46]. The priority of emotional memory over neutral information retention is mediated by noradrenergic activation, which is related to enhancement of the encoding of memories of traumatic events and can predict the severity of subsequent symptoms of posttraumatic stress disorder [47, 48]. Propranolol was shown to exert its effects by blocking β-adrenergic receptors in memory-related neurocircuits [37], thus preventing the modulatory influence of norepinephrine on the reconsolidation of emotional stimuli so that such stimuli are processed similarly to neutral stimuli. Propranolol may suppress the emotional valence of smoking memories, making the reactivation of smoking-related memories more similar to neutral memories. Consequently, the propranolol group may require the complementary activation of memory-related and reward-related brain regions to retrieve smoking memories. The increase in activation of the hippocampus in this study indicates that propranolol disrupted memory reconsolidation and deprived smoking-related cues of their rewarding properties by mediating the activation of memory-related brain regions.

However, several studies have proposed the boundary issue about memory reconsolidation interventions, that retrieval itself may not be enough to induced the reconsolidation process [49–51]. This study used the well-verified picture to induce memory retrieval, which is efficient to trigger smoking craving, and without nicotine intake, it created a mismatch and induced memory reconsolidation. In this study, we also detected decreased carving to smoking at baseline on day 3 after reconsolidation manipulation. One possible reason is that the propranolol not only disrupted the smoking memory reconsolidation, decreased the smoking cue-induced craving, but also decreased the general craving to smoking within the context of propranolol intake. Propranolol is thought to have an effect on blood pressure and heart rate, which may affect nicotine craving through decreasing the arousal level and ameliorating withdrawal symptoms in smokers. In addition to the theory of reconsolidation impairments, another alternative interpretation of the present findings is that propranolol itself may be a feasible treatment for addiction. However, evidence from previous studies does not support this interpretation. A previous study reported a weak effect of propranolol on nicotine-related cue-induced reinstatement of nicotine seeking in rodents [52]. There is no group difference in drug use outcomes after propranolol or placebo administration for 8 weeks in cocaine-dependent patients [53]. Moreover, another study found greater drug cue reactivity in propranolol-treated polydrug users (methadone-maintained opioid-dependent individuals who used cocaine) compared with those who received a placebo [54]. These results indicate that a single administration of propranolol does not affect maladaptive memory-based behavior [55–57]. Altogether, the hypothesis that propranolol itself attenuates craving for smoking instead of impairs reconsolidation appears to be inconsistent with existing data on propranolol’s efficacy in addiction.

This study has limitations. One key issue with the pharmacological manipulation of reconsolidation is the timing of propranolol administration. In this study, we did not investigate the time window of reconsolidation, which was assessed in our previous study. In previous studies [28, 58, 59], propranolol was administered before reactivation, similar to this study. A previous study found that propranolol intake 1 h before memory reactivation did not affect memory retrieval [60]. Another study also found that propranolol administration immediately after retrieval did not disrupt the reconsolidation of fear memory in humans [61]. Thus, in this study, the participants received propranolol before memory retrieval. But propranolol administration after nicotine memory retrieval should also be tested. Since medications were administered prior to both retrieval and putative memory destabilization, it is difficult to know with certainty the observed effects are due to medication effects on retrieval or reconsolidation. In most studies, successful memory retrieval is measured by skin conductance response or startle amplitude in fear memory. However, evidence has indicated a similar response of the CS+ for both groups at retrieval, which suggests that the retrieval process was not influenced by the administration of propranolol [60]. The present results are inconsistent with a previous study that administered a single dose of propranolol before memory reactivation and found no effect on craving for smoking 1 week after the intervention [62]. In the present study, we only tested the craving 24 h after the manipulation and not at longer time intervals, without follow-up data, it remains an open question whether the procedure was sufficient to treat smokers. In addition to subjective craving scores, we also evaluated associations between additional objective measurements, including changes in brain reactivity to smoking-related cues, and impairments in reconsolidation. There is no control for the possibility that any observed difference between the propranolol and placebo groups are due to propranolol exposure per se. However, according to several previous studies, the effect of propranolol alone on substance abuse has not been confirmed [26, 29, 58]. There is also a potential confound issue of cue habituation since the same cues were used across days and drug conditions, however, all of the participants went through the same procedure exactly, except for the drugs, and it is believed that the effect of cue habituation will not affect the group difference. The assessments of other substance use relied exclusively on self-report abstinence. Moreover, we only recruited male participants, so the results may not be representative of the population. Gender effects will need to be further explored in the future.

In summary, the present results showed that administration of the β-adrenergic receptor antagonist propranolol before memory reactivation blocked the re-stabilization of smoking-related cues and decreased craving in smokers by reorganizing the functional connectivity of memory-related brain regions, such as the hippocampus and striatum, during reconsolidation. Pre-retrieval propranolol administration also increased mPFC reactivity to smoking-related cues, which led to an increase in top-down regulation and a decrease in cue-induced craving. These findings highlight the ways in which propranolol-induced impairments in memory reconsolidation are represented in the human brain and the ways in which brain reactivity to smoking-related cues is influenced by the disruption of reconsolidation.

## Supplementary information

Supplemental material

## References

[1] WHO. Systematic review of the link between tobacco and poverty. Geneva, Switzerland: World Health Organization; 2011.

[2] WHO. WHO report on the global tobacco epidemic, 2015: raising taxes on tobacco. 2015;48:261–70.

[3] Torregrossa MM, Corlett PR, Taylor JR (2011). Aberrant learning and memory in addiction. Neurobiol Learn Mem.

[4] Berridge KC, Robinson TE (2016). Liking, wanting, and the incentive-sensitization theory of addiction. Am Psychol.

[5] Robinson TE, Berridge KC (1993). The neural basis of drug craving: an incentive-sensitization theory of addiction. Brain Res Brain Res Rev.

[6] Waters AJ, Shiffman S, Sayette MA, Paty JA, Gwaltney CJ, Balabanis MH (2003). Attentional bias predicts outcome in smoking cessation. Health Psychol.

[7] Carpenter MJ, Saladin ME, DeSantis S, Gray KM, LaRowe SD, Upadhyaya HP (2009). Laboratory-based, cue-elicited craving and cue reactivity as predictors of naturally occurring smoking behavior. Addict Behav.

[8] Nader K, Schafe GE, Le Doux JE (2000). Fear memories require protein synthesis in the amygdala for reconsolidation after retrieval. Nature.

[9] Vallejo AG, Kroes MC, Rey E, Acedo MV, Moratti S, Fernández G (2019). Propofol-induced deep sedation reduces emotional episodic memory reconsolidation in humans. Sci Adv.

[10] Auchter AM, Shumake J, Gonzalez-Lima F, Monfils MH (2017). Preventing the return of fear using reconsolidation updating and methylene blue is differentially dependent on extinction learning. Sci Rep..

[11] Elsey J, Filmer AI, Galvin HR, Kurath JD, Vossoughi L, Thomander LS (2020). Reconsolidation-based treatment for fear of public speaking: a systematic pilot study using propranolol. Transl Psychiatry.

[12] Fan HY, Cherng CG, Yang FY, Cheng LY, Tsai CJ, Lin LC (2010). Systemic treatment with protein synthesis inhibitors attenuates the expression of cocaine memory. Behav Brain Res.

[13] Valjent E, Corbille AG, Bertran-Gonzalez J, Herve D, Girault JA (2006). Inhibition of ERK pathway or protein synthesis during reexposure to drugs of abuse erases previously learned place preference. Proc Natl Acad Sci USA.

[14] Sorg BA (2012). Reconsolidation of drug memories. Neurosci Biobehav Rev.

[15] Thonberg H, Fredriksson JM, Nedergaard J, Cannon B (2002). A novel pathway for adrenergic stimulation of cAMP-response-element-binding protein (CREB) phosphorylation: mediation via alpha1-adrenoceptors and protein kinase C activation. Biochem J.

[16] Elsey JWB, Kindt M (2017). Tackling maladaptive memories through reconsolidation: from neural to clinical science. Neurobiol Learn Mem.

[17] Lonergan MH, Olivera-Figueroa LA, Pitman RK, Brunet A (2013). Propranolol’s effects on the consolidation and reconsolidation of long-term emotional memory in healthy participants: a meta-analysis. J Psychiatry Neurosci.

[18] Dębiec J, Ledoux JE (2004). Disruption of reconsolidation but not consolidation of auditory fear conditioning by noradrenergic blockade in the amygdala. Neuroscience..

[19] Brunet A, Saumier D, Liu A, Streiner DL, Tremblay J, Pitman RK (2018). Reduction of PTSD symptoms with pre-reactivation propranolol therapy: a randomized controlled trial. Am J Psychiatry.

[20] Mahabir M, Tucholka A, Shin LM, Etienne P, Brunet A (2015). Emotional face processing in post-traumatic stress disorder after reconsolidation impairment using propranolol: a pilot fMRI study. J Anxiety Disord.

[21] Levy DA, Mika R, Radzyminski C, Ben-Zvi S, Tibon R (2018). Behavioral reconsolidation interference with episodic memory within-subjects is elusive. Neurobiol Learn Mem.

[22] Schroyens N, Beckers T, Kindt M (2017). In search for boundary conditions of reconsolidation: a failure of fear memory interference. Front Behav Neurosci.

[23] Lonergan M, Saumier D, Tremblay J, Kieffer B, Brown TG, Brunet A (2016). Reactivating addiction-related memories under propranolol to reduce craving: a pilot randomized controlled trial. J Behav Ther Exp Psychiatry.

[24] Robinson MJF, Franklin KBJ (2007). Central but not peripheral beta-adrenergic antagonism blocks reconsolidation for a morphine place preference. Behav Brain Res.

[25] Taylor JR, Olausson P, Quinn JJ, Torregrossa MM (2009). Targeting extinction and reconsolidation mechanisms to combat the impact of drug cues on addiction. Neuropharmacology..

[26] Saladin ME, Gray KM, McRae-Clark AL, Larowe SD, Yeatts SD, Baker NL (2013). A double blind, placebo-controlled study of the effects of post-retrieval propranolol on reconsolidation of memory for craving and cue reactivity in cocaine dependent humans. Psychopharmacology..

[27] Zhao LY, Sun LL, Shi J, Li P, Zhang Y, Lu L (2011). Effects of β-adrenergic receptor blockade on drug-related memory reconsolidation in abstinent heroin addicts. Drug Alcohol Depend.

[28] Xue YX, Deng JH, Chen YY, Zhang LB, Wu P, Huang GD (2017). Effect of selective inhibition of reactivated nicotine-associated memories with propranolol on nicotine craving. JAMA Psychiatry.

[29] Schwabe L, Nader K, Wolf OT, Beaudry T, Pruessner JC (2012). Neural signature of reconsolidation impairments by propranolol in humans. Biol Psychiatry.

[30] Schiller D, Kanen JW, LeDoux JE, Monfils MH, Phelps EA (2013). Extinction during reconsolidation of threat memory diminishes prefrontal cortex involvement. Proc Natl Acad Sci USA.

[31] Agren T, Engman J, Frick A, Björkstrand J, Larsson EM, Furmark T (2012). Disruption of reconsolidation erases a fear memory trace in the human amygdala. Science.

[32] Björkstrand J, Agren T, Frick A, Engman J, Larsson EM, Furmark T (2015). Disruption of memory reconsolidation erases a fear memory trace in the human amygdala: an 18-month follow-up. PLoS ONE.

[33] Björkstrand J, Agren T, Åhs F, Frick A, Larsson EM, Hjorth O (2016). Disrupting reconsolidation attenuates long-term fear memory in the human amygdala and facilitates approach behavior. Curr Biol: CB.

[34] Kroes MC, Tona KD, den Ouden HE, Vogel S, van Wingen GA, Fernández G (2016). How administration of the beta-blocker propranolol before extinction can prevent the return of fear. Neuropsychopharmacology..

[35] Mohseni M (2013). Use of vitamin C as placebo in anesthesiology. Anesth Pain Med.

[36] Deng J, Shi L, Yuan K, Yao P, Chen S, Que J (2020). Propranolol-induced inhibition of unconditioned stimulus-reactivated fear memory prevents the return of fear in humans. Transl Psychiatry.

[37] Kindt M, Soeter M, Vervliet B (2009). Beyond extinction: erasing human fear responses and preventing the return of fear. Nat Neurosci.

[38] Paterson JW, Conolly ME, Dollery CT, Hayes A, Cooper RG (1970). The pharmacodynamics and metabolism of propranolol in man. Pharmacologia Clin.

[39] Rao L-L, Zhou Y, Xu L, Liang ZY, Jiang T, Li S (2011). Are risky choices actually guided by a compensatory process? new insights from fMRI. PLoS ONE.

[40] Wang Y, Zhong S, Jia Y, Sun Y, Wang B, Liu T (2016). Disrupted resting-state functional connectivity in nonmedicated bipolar disorder. Radiology..

[41] Rao L-L, Dunn JC, Zhou Y, Li S (2015). The neural correlates of risky decision making across short and long runs. Sci Rep..

[42] Kuhn S, Gallinat J (2011). Common biology of craving across legal and illegal drugs - a quantitative meta-analysis of cue-reactivity brain response. Eur J Neurosci.

[43] Jasinska AJ, Stein EA, Kaiser J, Naumer MJ, Yalachkov Y (2014). Factors modulating neural reactivity to drug cues in addiction: a survey of human neuroimaging studies. Neurosci Biobehav Rev.

[44] Franklin TR, Wang Z, Li Y, Suh JJ, Goldman M, Lohoff FW (2011). Dopamine transporter genotype modulation of neural responses to smoking cues: confirmation in a new cohort. Addict Biol.

[45] Hartwell KJ, Johnson KA, Li X, Myrick H, LeMatty T, George MS (2011). Neural correlates of craving and resisting craving for tobacco in nicotine dependent smokers. Addict Biol.

[46] Ferry B, Roozendaal B, McGaugh JL (1999). Basolateral amygdala noradrenergic influences on memory storage are mediated by an interaction between beta- and alpha1-adrenoceptors. J Neurosci.

[47] O’Donnell T, Hegadoren KM, Coupland NC (2004). Noradrenergic mechanisms in the pathophysiology of post-traumatic stress disorder. Neuropsychobiology..

[48] Vaiva G, Ducrocq F, Jezequel K, Averland B, Lestavel P, Brunet A, Marmar CR (2003). Immediate treatment with propranolol decreases posttraumatic stress disorder two months after trauma. Biol Psychiatry.

[49] Auber A, Tedesco V, Jones CE, Monfils M-H, Chiamulera C (2013). Post-retrieval extinction as reconsolidation interference: methodological issues or boundary conditions?. Psychopharmacology..

[50] Walker MP, Stickgold R (2016). Understanding the boundary conditions of memory reconsolidation. Proc Natl Acad Sci USA.

[51] Zuccolo PF, Hunziker MHL (2019). A review of boundary conditions and variables involved in the prevention of return of fear after post-retrieval extinction. Behav Process.

[52] Chiamulera C, Tedesco V, Zangrandi L, Giuliano C, Fumagalli G (2010). Propranolol transiently inhibits reinstatement of nicotine-seeking behaviour in rats. J Psychopharmacol.

[53] Kampman KM, Volpicelli JR, Mulvaney F, Alterman AI, Cornish J, Gariti P (2001). Effectiveness of propranolol for cocaine dependence treatment may depend on cocaine withdrawal symptom severity. Drug Alcohol Depend.

[54] Jobes ML, Aharonovich E, Epstein DH, Phillips KA, Reamer D, Anderson M, Preston KL (2015). Effects of prereactivation propranolol on cocaine craving elicited by imagery script/cue sets in opioid-dependent polydrug users: a randomized study. J Addict Med.

[55] Rodriguez-Romaguera J, Sotres-Bayon F, Mueller D, Quirk GJ (2009). Systemic propranolol acts centrally to reduce conditioned fear in rats without impairing extinction. Biol Psychol.

[56] Bos MG, Beckers T, Kindt M (2012). The effects of noradrenergic blockade on extinction in humans. Biol Psychol.

[57] Brunet A, Orr SP, Tremblay J, Robertson K, Nader K, Pitman RK (2008). Effect of post-retrieval propranolol on psychophysiologic responding during subsequent script-driven traumatic imagery in post-traumatic stress disorder. J Psychiatr Res.

[58] Thomas É, Saumier D, Pitman RK, Tremblay J, Brunet A (2017). Consolidation and reconsolidation are impaired by oral propranolol administered before but not after memory (re)activation in humans. Neurobiol Learn Mem.

[59] Kindt M, Soeter M (2018). Pharmacologically induced amnesia for learned fear is time and sleep dependent. Nat Commun.

[60] Soeter M, Kindt M (2012). Stimulation of the noradrenergic system during memory formation impairs extinction learning but not the disruption of reconsolidation. Neuropsychopharmacology..

[61] Thomas E, Saumier D, Pitman RK, Tremblay J, Brunet A (2017). Consolidation and reconsolidation are impaired by oral propranolol administered before but not after memory (re)activation in humans. Neurobiol Learn Mem.

[62] Pachas GN, Gilman J, Orr SP, Hoeppner B, Carlini SV, Grasser EB (2015). Single dose propranolol does not affect physiologic or emotional reactivity to smoking cues. Psychopharmacology.

