# Supplementary material for: Neural substrates of propranolol-induced impairments in the reconsolidation of nicotine-associated memories in smokers
Source: Transl Psychiatry. 2021 Aug 24;11:441. doi: 10.1038/s41398-021-01566-6 (PMC8385067; doi:10.1038/s41398-021-01566-6)
Supplement: Supplementary file 1 — Supplemental material [file 41398_2021_1566_MOESM1_ESM.docx]

March 7, 2021, Aritical for *Translational Psychiatry* (No. 2020TP001107R)

**SUPPLEMENTARY MATERIAL**

**Neural substrates of** **propranolol-induced impairments in the reconsolidation of nicotine-associated memories in smokers**

**Xiao Lin,^1, #^ Jiahui Deng,^1, #^ Kai Yuan,^1, #^ Qiandong Wang,^2^ Lin Liu,^1^ Yanping Bao,^3^ Yanxue Xue,^3^ Peng Li,^1^ Jianyu Que,^1^ Jiajia Liu,^1^ Wei Yan,^1^ Hongqiang Sun,^1^ Ping Wu,^3^ Jie Shi,^3^ Le Shi,^1, *^ Lin Lu^1, 3, 4 ,*^**

^1^ Peking University Sixth Hospital, Peking University Institute of Mental Health, NHC Key Laboratory of Mental Health (Peking University), National Clinical Research Center for Mental Disorders (Peking University Sixth Hospital), Chinese Academy of Medical Sciences Research Unit (No.2018RU006), Peking University, Beijing 100191, China

^2^ Beijing Key Laboratory of Applied Experimental Psychology, National Demonstration Center for Experimental Psychology Education (Beijing Normal University), Faculty of Psychology, Beijing Normal University, Beijing 100875, China.

^3^ National Institute on Drug Dependence and Beijing Key Laboratory on Drug Dependence Research, Peking University, Beijing 100191, China

^4^ Peking-Tsinghua Center for Life Sciences and PKU-IDG/McGovern Institute for Brain Research, Beijing 100191, China

^#^These authors contributed equally to this work

*Corresponding author:

Lin Lu, MD, PhD, Institute of Mental Health and Peking University Sixth Hospital, 51 Huayuanbei Road, Beijing, 100191, China.

Tel: +86-10-82805308; Fax: +86-10-62032624; E-mail: [linlu@bjmu.edu.cn](mailto:linlu@bjmu.edu.cn)

OR

Le Shi, PhD, Institute of Mental Health and Peking University Sixth Hospital, 51 Huayuanbei Road, Beijing, 100191, China.

Tel: +86-10-82803704; Fax: +86-10-62032624; E-mail: leshi@bjmu.edu.cn

**Manuscript information:** 5 figures, 3 tables, 4749 words

**Running title:** Propranolol disrupts smoking memory reconsolidation

**Supplementary Methods**

*Cue reactivity task*

In each trial, three pictures appeared on the screen for 2 s, with one on the top of the screen and two at the bottom. The participants were required to press a key (left key or right key) to indicate which picture at the bottom was same as the top picture. There were 24 smoking-related pictures and 24 neutral pictures. Each picture was presented twice in the scanner, for a total of 96 trials. For each trial, the reaction time was limited to 4 s. Once the participant made a response, the screen went black until 4 s elapsed for each trial. Timeout trials were not included in the statistical analysis.

*Three-way ANOVA results*

A three-way ANOVA, with time (day 1, day 3), cue type (smoking-related cues, neutral cues), and group (propranolol, placebo) as factors, was conducted to analyze the effect of the reconsolidation manipulation on brain changes in response to smoking-related cues.

**
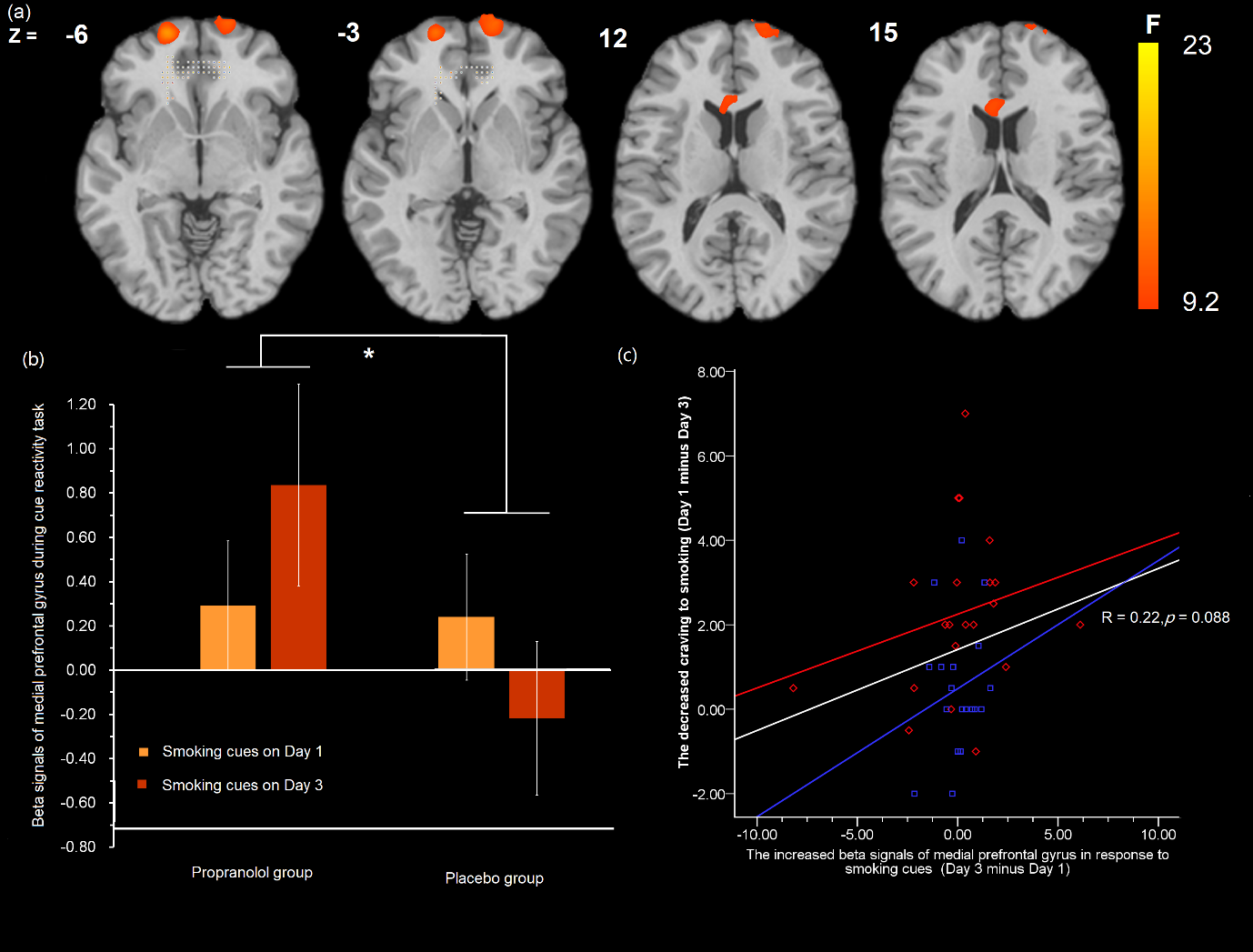
**

**Figure S1. a.** Significant group (propranolol, placebo) × time (day 1, day 3) × cue type (smoking-related cues, neutral cues) interaction in the three-way repeated-measures ANOVA, showing that the peak brain regions were the prefrontal cortex (mPFC) and the anterior cingulate cortex (ACC). **b.** Parameter estimates of interactive effect cluster (mPFC) were significantly increased to smoking cues in the propranolol group compared to the placebo group. The difference was only significant on day 3 (i.e., 1 days after reconsolidation impairment). **c.** Positive correlation between increased mPFC activity and decreased craving from Day 1 to Day3. The correlation was marginal significant. Error bars represent the SEM. **p* < 0.05.


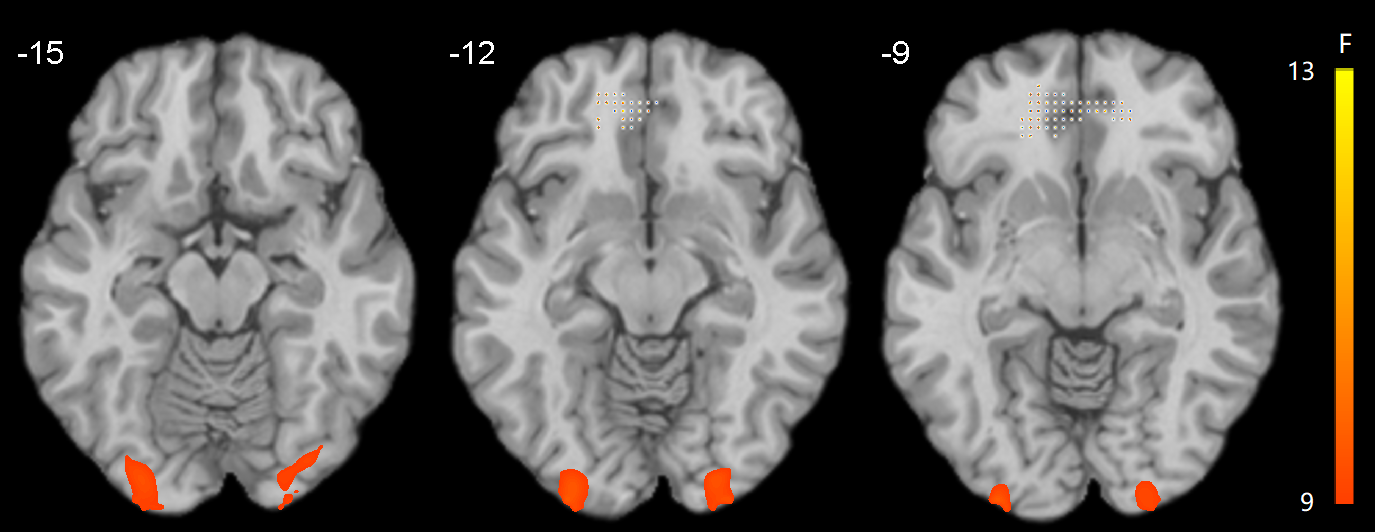


**Figure S2.** Brain regions that showed a main effect of group in the three-way repeated-repeated ANOVA during the cue reactivity task before and after propranolol administration.

Table S1. Clusters showed significantly interactions in the three-way ANOVA (group × picture type × time).

| **Anatomical region** |  | **Coordinates** |  | **F** | **Cluster size** | **Hemisphere** | **Brodmann area** |
| --- | --- | --- | --- | --- | --- | --- | --- |
| **Interactions (group × picture type × time)** | X | Y | Z |  |  |  |  |
| Anterior cingulate gyrus | -4 | 21 | 18 | 13.255 | 32 | L | 33 |
| Superior Frontal Gyrus | -22 | 60 | -7 | 11.668 | 37 | L | 10 |
| Medial Frontal Gyrus | 19 | 63 | -4 | 10.201 | 107 | R | 10 |

Table S2. Clusters that showed a main effect of group in the three-way ANOVA.

| **Anatomical region** | |  | **Coordinates** |  | **F** | **Cluster size** | **Hemisphere** | **Brodmann area** |
| --- | --- | --- | --- | --- | --- | --- | --- | --- |
| **Main effect of group** |  | X | Y | Z |  |  |  |  |
| Inferior Occipital Gyrus | | -29 | -93 | -6 | 10.85025 | 51 | L | 18 |
| Inferior Occipital Gyrus | | 24 | -88 | -7 | 10.40356 | 34 | R | 18 |

Coordinates are given for the maximally significant voxel in each area, where X defines the lateral placement from the midline (left = negative), Y defines the anteroposterior displacement relative to the anterior commissure (posterior = negative), and Z defines the vertical position relative to the anteroposterior commissural line (down = negative). *p* values were corrected for multiple comparisons at *p* < 0.005 (alphasim corrected). The coordinates are in Montreal Neurological Institute (MNI) space. The brain regions were automatically identified by NeuroElf software.
